# Supplementary material for: Causal Relationship between Aging and Anorexia Nervosa: A White-Matter-Microstructure-Mediated Mendelian Randomization Analysis
Source: Biomedicines. 2024 Aug 16;12(8):1874. doi: 10.3390/biomedicines12081874 (PMC11351342; doi:10.3390/biomedicines12081874)
Supplement: Supplementary file 1 [file biomedicines-12-01874-s001.zip › Supplementary Figure/Figure S4/Singe SNP plot of MR analysis for TL to ubm-b-2008.pdf]

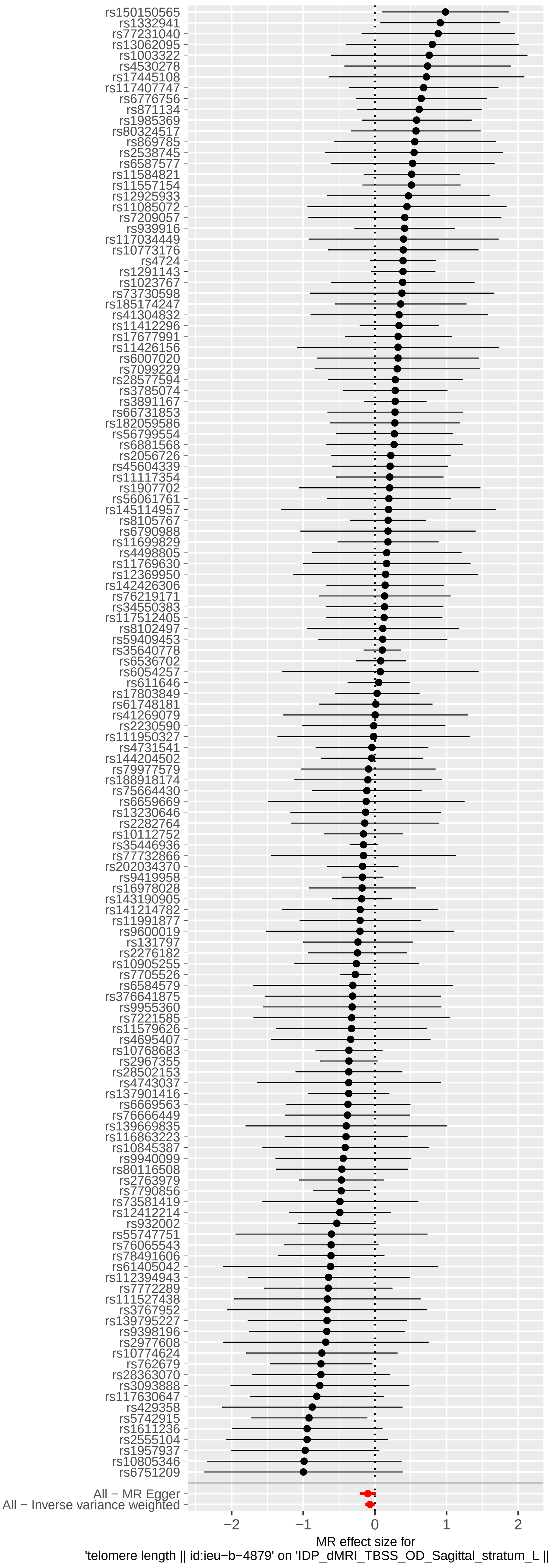

rs150150565
rs1332941
rs77231040
rs13062095
rs1003322
rs4530278
rs17445108
rs117407747
rs6776756
rs871134
rs1985369
rs80324517
rs869785
rs2538745
rs6587577
rs11584821
rs11557154
rs12925933
rs11085072
rs7209057
rs939916
rs117034449
rs10773176
rs4724
rs1291143
rs1023767
rs73730598
rs185174247
rs41304832
rs11412296
rs17677991
rs11426156
rs6007020
rs7099229
rs28577594
rs3785074
rs3891167
rs66731853
rs182059586
rs56799554
rs6881568
rs2056726
rs45604339
rs11117354
rs1907702
rs56061761
rs145114957
rs8105767
rs6790988
rs11699829
rs4498805
rs11769630
rs12369950
rs142426306
rs76219171
rs34550383
rs117512405
rs8102497
rs59409453
rs35640778
rs6536702
rs6054257
rs611646
rs17803849
rs61748181
rs41269079
rs2230590
rs111950327
rs4731541
rs144204502
rs79977579
rs188918174
rs75664430
rs6659669
rs13230646
rs2282764
rs10112752
rs35446936
rs77732866
rs202034370
rs9419958
rs16978028
rs143190905
rs141214782
rs11991877
rs9600019
rs131797
rs2276182
rs10905255
rs7705526
rs6584579
rs376641875
rs9955360
rs7221585
rs11579626
rs4695407
rs10768683
rs2967355
rs28502153
rs4743037
rs137901416
rs6669563
rs76666449
rs139669835
rs116863223
rs10845387
rs9940099
rs80116508
rs2763979
rs7790856
rs73581419
rs12412214
rs932002
rs55747751
rs76065543
rs78491606
rs61405042
rs112394943
rs7772289
rs111527438
rs3767952
rs139795227
rs9398196
rs2977608
rs10774624
rs762679
rs28363070
rs3093888
rs117630647
rs429358
rs5742915
rs1611236
rs2555104
rs1957937
rs10805346
rs6751209
All − MR Egger
All − Inverse variance weighted
−2
−1
0
1
2
MR effect size for
'telomere length || id:ieu−b−4879' on 'IDP_dMRI_TBSS_OD_Sagittal_stratum_L ||
